# Navigating Anesthetic Challenges in Multiple Spinal Hydatid Cysts

**DOI:** 10.7759/cureus.66725

**Published:** 2024-08-12

**Authors:** Chhaya M Suryawanshi, Jayant Bhatia

**Affiliations:** 1 Anesthesiology, Dr. D.Y. Patil Medical College, Hospital and Research Centre, Dr. D.Y. Patil Vidyapeeth (Deemed to be University), Pune, IND

**Keywords:** laminectomy, spinal compression, hydatid cyst, echinococcus granulosus, anaphylaxis

## Abstract

Spinal hydatid disease is a rare form of hydatid disease caused by the larval stage of Echinococcus granulosus. It refers to a range of conditions that affect the spinal cord, the spine, or both. The prevalence of spinal hydatid disease is highest in the thoracic spine; however, it can also occur in other regions of the spine.

In this case report, we present an unusual occurrence of numerous hydatid cysts in a 42-year-old male living in a remote region. The patient has been experiencing a progressive loss of power in his lower limbs, urine incontinence, and back pain for the past four months. The patient was found to have many distinct cystic lesions with spinal cord compression syndrome. Spinal hydatidosis is an uncommon illness that causes significant suffering and has a bleak outlook. When evaluating a patient with spinal compression syndrome, it is important to evaluate this as one of the potential causes.

## Introduction

Hydatid cysts are a parasitic infection caused by the larvae of Echinococcus granulosus cestode [1]. This disease is more widespread in locations where cattle and agriculture are prevalent. The fatality rate for this illness varies from 0.9% to 3.6% [2]. The infection has the potential to impact all organs and tissues, with approximately 60%-70% of hydatid cysts developing in the liver and 15%-20% in the lungs. Bone involvement is infrequent, with a prevalence of approximately 0.5%-2% in instances, and over half of these cases specifically impact the vertebrae [3]. Spinal cord involvement is exceptionally uncommon, occurring in about 1% of cases, and it usually has a negative outlook. It most commonly affects the thoracic vertebrae [4].

The clinical signs are typically nonspecific and latent, and they vary depending on the location and size of the lesions. Signs of the condition usually appear as the cysts grow in size and can vary from nerve pain to bone fractures. Spinal hydatid cysts can appear in five specific locations: intramedullary, intradural extramedullary, extradural, vertebral, and paravertebral lesions [5]. The main therapeutic approach for spinal echinococcosis is surgery [6]. Avoiding biopsy or aspiration in cases where there is suspicion of this infection is paramount, as it carries a risk of anaphylactic reactions. This case represents a comprehensive analysis of spinal hydatidosis.

## Case presentation

A 42-year-old male from a rural area was admitted to the Neurosurgery ward with complaints of progressive lower limb weakness, urinary incontinence, and lumbar back pain for four months. The patient had a history of back pain for the past 10 years, which had now aggravated over the past four months.

The patient lacked any pets and had no prior documented allergies. He underwent a T6 to T10 laminectomy 15 years ago due to back pain and lower limb paralysis. The procedure involved the removal of multiloculated fluid-filled, non-enhancing intramural extramedullary lesions, which were subsequently diagnosed as hydatid cysts. After a period of two years, the individual experienced repeated episodes of back discomfort, as well as a sensation of tingling and loss of feeling in both legs. Neuroimaging techniques revealed the presence of recurrent intraspinal lesions that extended from the T6 to the T10 level. A second surgical procedure was performed in order to eliminate the recurrent lesions. One year later, the individual experienced tingling sensations in both feet and numbness extending from the waist to the ankles, marking the third occurrence of these symptoms.

**Figure 1:**
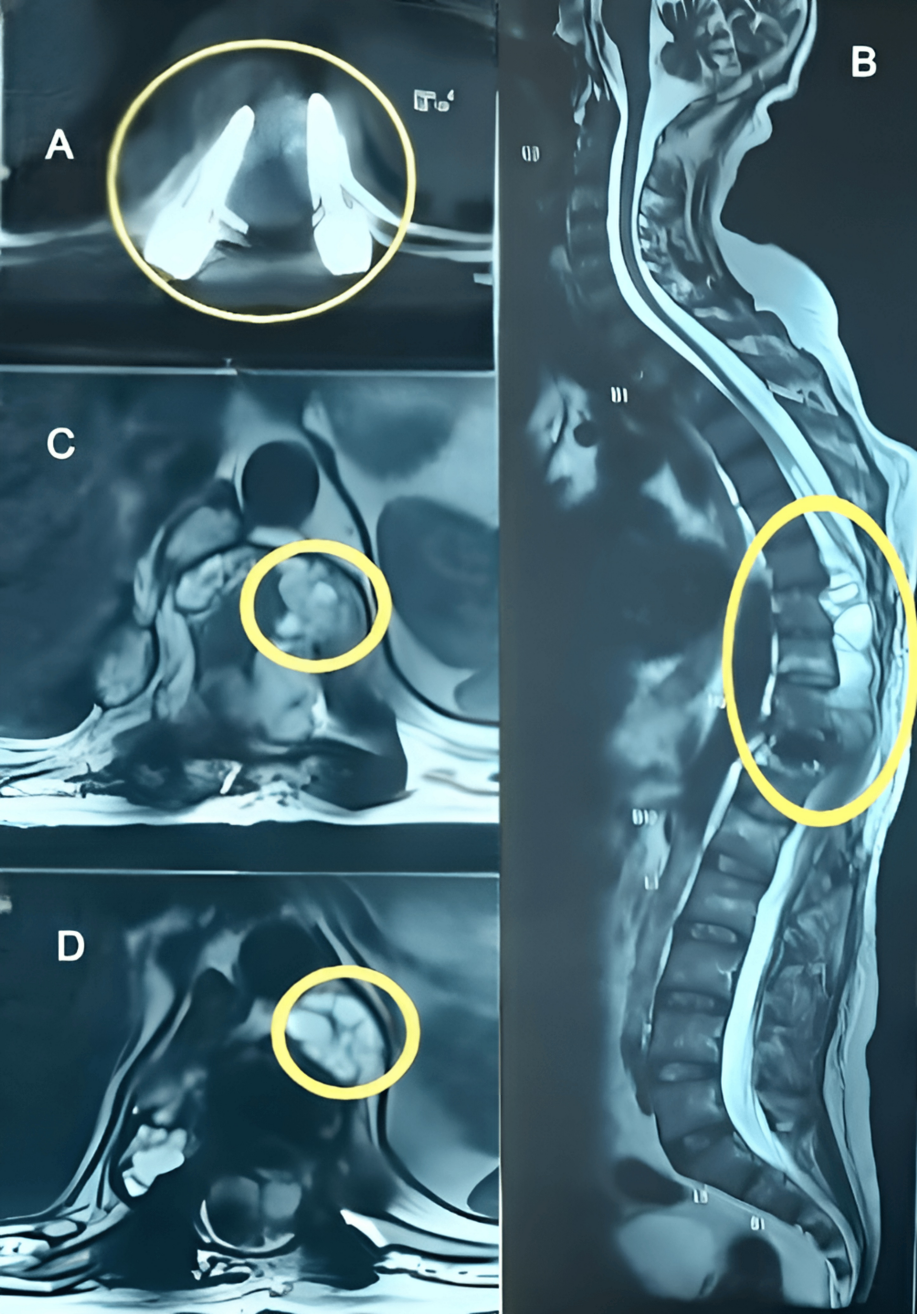
A: Transpedicular Screw Fixation, B: Fluid-filled Lesions, C: Cystic Lesions adjacent to T9, and D: Cystic Lesions adjacent to T10

Magnetic resonance imaging (MRI) revealed post-operative changes, specifically a laminectomy at the T6 to T10 vertebral levels along with the use of transpedicular fixation screws to stabilize the T7 to L1 vertebrae (Figure 1A). Fluid-filled cysts were found within the spinal canal, outside the spinal cord, between the T6 and T9 vertebrae (Figure 1B). These cysts measured 9.8x3x2.3 cm in antero-posterior dimensions. Additionally, there were cystic lesions near the T9 (Figure 1C) and T10 (Figure 1D) vertebrae, along with bilateral neural foramina at various levels. The presence of these lesions caused the spinal cord to be displaced toward the back and compressed over a significant length, notably between the T6 and T10 vertebrae. Additionally, there were swelling and edema observed in the lower thoracic spinal cord at the T11 and T12 levels.

The patient was commenced on Tab. Albendazole 400 mg twice daily and a surgical procedure was arranged to remove the cysts using the posterior technique, in order to relieve the compression, while under general anesthesia.

During the pre-anesthetic examination, the patient was in a satisfactory overall state, with a blood pressure of 120/70 mmHg, a heart rate of 80 beats per minute, and a peripheral oxygen saturation (SpO_2_) of 99% on room air. The patient's cardiovascular examination indicated normal heart sounds without any murmurs. During the examination of the respiratory system, there was symmetrical and equal air entry on both sides, without any abnormal sounds. The neurological examination showed a Glasgow Coma Scale (GCS) score of 15/15. However, there was flaccid paraplegia, with a loss of muscle tone and function in both lower limbs. The power in the upper limbs was 5/5, indicating normal strength, while the power in the lower limbs was 0/5, indicating complete loss of strength. Additionally, there was hypoesthesia, below the T10 level. The abdominal, patellar, and ankle reflexes were absent with extensor plantar responses.

The patient's preoperative bloodwork was all within the normal range. A recent chest X-ray revealed no abnormalities, and the electrocardiography (ECG) indicated a normal sinus rhythm. The 2D-Echocardiography revealed a normal left ventricular ejection fraction (LVEF) of 60%. In light of all normal investigations, the patient was granted anesthesia fitness under American Society of Anesthesiologists (ASA) grade II with high-risk, post-operative Intensive Care Unit (ICU) and ventilator consent.

In the operating room, traditional monitoring devices, including a blood pressure cuff, ECG, a temperature probe, and a SpO_2_ probe, were put in. Ringer's Lactate IV fluid was initiated at a rate of 10-20 ml/kg after a 16G peripheral intravenous cannula was secured to the right hand. A cover for anaphylaxis was administered with 8 mg of dexamethasone intravenously.

The patient was subjected to 100% oxygenation for a duration of three minutes prior to the induction. During the induction of general anesthesia, the following medications were delivered intravenously: 2 mg/kg of propofol, 2 mcg/kg of fentanyl, and 0.1 mg/kg of vecuronium. The patient had orotracheal intubation using an 8.5 flexometallic endotracheal tube. This was followed by assessing chest rise during ventilation, monitoring the presence of end-tidal CO2 (EtCO2), listening for breath sounds on both sides of the chest, and confirming the correct placement of the tube. In the right internal jugular vein, a Centro-Romsons® 7-Fr triple-lumen central venous catheter was put in, and a 20G IV cannula was used to cannulate the left radial artery. The patient was then meticulously placed in a prone position with supportive padding to prevent injuries to the abdomen and thorax. Proper eye padding was also applied. The respiratory rate was calibrated to maintain a normal end-tidal carbon dioxide (EtCO2) level, and the ventilator was configured to operate in volume control mode with a tidal volume (TV) of 7 ml per kilogram. We used a low-flow combination for maintenance, 1000 mL/minute total fresh gas flow, consisting of 50% oxygen, 50% nitrogen, and isoflurane (with a minimum alveolar concentration of 1).

Emergency medications such as Inj. Atropine, Inj. Adrenaline, Inj. Dexamethasone, Inj. Hydrocortisone, Inj. Mephentermine, Inj. Soda bicarbonate, Inj. Deriphylline, and Salbutamol inhaler were readily accessible in the operating room as a precautionary measure to address an anaphylactic reaction, should it occur during the removal of the hydatid cyst.

During the surgery, the patient's blood pressure remained stable despite 550ml of blood loss. The intravenous fluid therapy during the five-hour surgery included 500ml of Hydroxyethyl starch (Voluven®) and 300ml of packed red cell volume as colloids, as well as 1000ml of Ringer's Lactate and 500ml of normal saline as crystalloids. The objective was to maintain adequate urine output (0.5-1 ml/kg/hour) and keep the central venous pressure between 8 and 10 mmHg. A single intravenous injection of 100mg hydrocortisone was administered immediately, followed by the careful removal of the cysts, taking essential measures to prevent any leakage of the cyst fluid into the surrounding structures.

**Figure 2:**
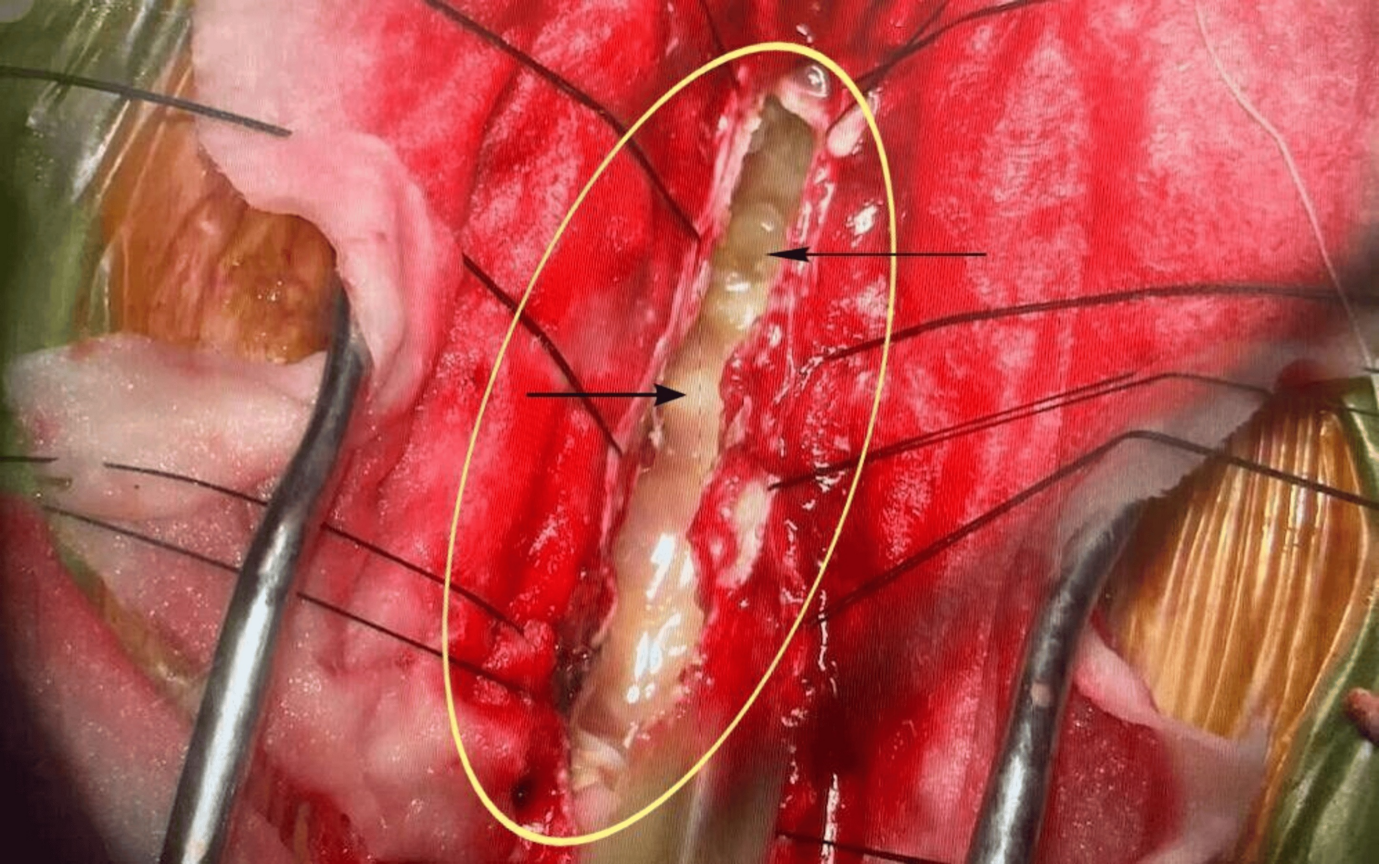
Multiple hydatid cysts

**Figure 3:**
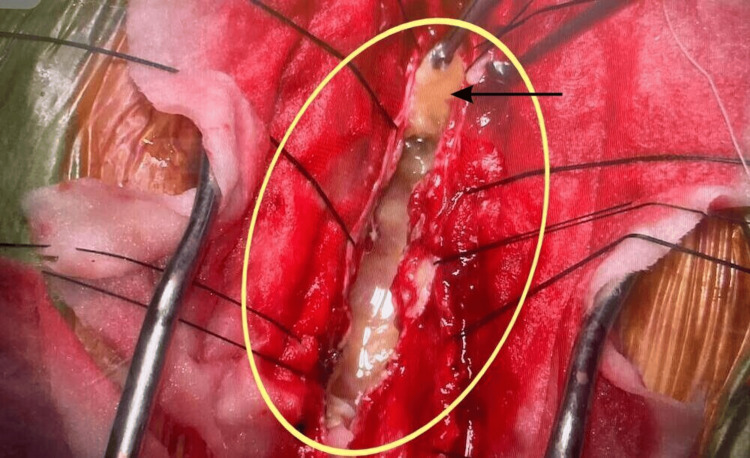
Removal of hydatid cysts

The configuration of the lesion (Figures 2, 3) posed a risk of rupture, tissue contamination, and anaphylaxis during in situ, en bloc removal.

Following the surgery, the patient's position was changed back to lying on their back and was administered Inj. Neostigmine 0.05 mg/kg and Inj. Glycopyrrolate 0.008 mg/kg to counteract the effects of anesthesia. The patient was subsequently and successfully extubated without encountering any immediate respiratory complications like stridor, bronchospasm, laryngospasm, or any respiratory distress. The patient experienced no pain and had stable vital signs. Consequently, the patient was moved to the ICU on oxygen at a rate of 6 liters per minute through an oxygen face mask. The patient received postoperative pain relief with intravenous administration of 1 gram of paracetamol every eight hours and a 2.5mg Fentanyl transdermal patch was applied the night before the surgery, delivering a dose of 25mcg/hour.

The patient's postoperative course was uneventful, with gradual improvement in his neurological deficits. The patient was then discharged after 10 days without any neurological deficits. Post-operatively, Inj. Dexamethasone 8 mg OD was continued which was then tapered off to 4 mg OD and thereafter stopped on Day 8. Later, albendazole at a dose of 15 mg/kg/day was continued for three months, and the patient was advised to follow up for further management and monitoring.

## Discussion

Echinococcosis is a persistent infectious disease that is transmitted to humans through food infected with eggs of the Echinococcus spp. tapeworm. This illness is widespread and mostly found in tropical and subtropical areas. The extensive dispersion of the virus poses difficulties in precisely assessing the prevalence and count of infected persons. Humans act as intermediate hosts in the life cycle of echinococcosis, while carnivores serve as definitive hosts and the main source of infection [7]. In India, agricultural techniques entail the utilization of physical labor in the cultivation of land and close interaction with cattle. Soil contamination caused by contaminated feces and subsequent eating can result in the contraction of the disease. The oncospheres released from the eggs in the colon enter the portal circulation, typically impacting the liver, yet additionally affecting other locations by transmitting blood-borne pathogens [8].

During excisional surgery, the rupture of a hydatid cyst might trigger an allergic reaction, which can result in death due to complications connected to anaphylaxis. The occurrence of intraoperative anaphylaxis caused by the rupture of hydatid cysts is estimated to range from 0.2% to 3.3%. This reaction is triggered by a type 1 hypersensitivity response mediated by IgE antibodies.

Anaphylaxis is a severe and sometimes fatal systemic reaction that can impact various organs [9]. Anaphylaxis occurring under anesthesia can manifest in several ways. The symptoms and signs of this condition, which are consistent with anaphylactic reactions in general, may be concealed by factors such as low blood volume, the level of anesthesia (light or deep), or the use of extensive regional anesthesia. Common cutaneous symptoms, such as flushing, urticaria, and edema, are typically concealed by surgical drapes during anesthesia. Cardiovascular symptoms commonly include low blood pressure and rapid heart rate but can quickly escalate to serious irregular heart rhythms and a sudden failure of the cardiovascular system if not identified and addressed promptly. The symptoms mentioned are both prevalent and severe, and in certain instances, the only symptom that may be observed is cardiovascular collapse. Respiratory symptoms, such as bronchospasm, following the administration of anesthesia are rather infrequent but may be more prevalent in individuals who already have asthma [10]. The fatality rate for anaphylaxis varies from 3% to 6% [11].

Anaphylaxis occurring under anesthesia exhibits a diverse range of manifestations, and the appropriate course of action for therapy will invariably be determined by the specific clinical presentation. Adrenaline and IV fluid therapy are the fundamental components of treatment. Adrenaline is a highly efficient and successful treatment for anaphylaxis in the majority of cases. It is recommended to provide the medication as soon as possible and adjust the dosage carefully based on the patient's response, particularly when given intravenously (at a dose of 0.1-1mg). The α-agonist property reduces vasodilation and edema, while the β-agonist property expands the airways, improves cardiac output, and reduces the synthesis of inflammatory mediators including leukotrienes and histamine [12]. Fluid therapy is crucial for mitigating the significant changes in fluid distribution caused by vasodilation and leaking of capillaries. In extreme instances, several liters of crystalloid/colloid solutions are required.

Secondary treatment for anaphylaxis includes the use of corticosteroids and antihistamines. These medications help avoid swelling, skin complaints, and recurrence of the anaphylactic reaction, which may happen within 24 hours after the initial reaction [13]. Therefore, it is crucial to carefully examine the extent of monitoring and observation required for the patient after successfully treating an anaphylactic reaction.

In the event of anaphylaxis, it is imperative to promptly cease all drugs and surgical procedures and supply 100% oxygen. Epinephrine is the initial treatment for anaphylaxis that occurs during surgery, as it acts as a vasopressor. Glucocorticoids are used to reduce the delayed symptoms of the allergic reaction.

The intraoperative, posterior midline approach is the standard surgical approach for accessing spine lesions, as it provides adequate exposure while minimizing cord manipulation. For this reason, prone positioning is commonly utilized for access during spinal surgery [14]. Nevertheless, assuming this position increases the pressure in the chest cavity, leading to a reduction in the amount of blood returning to the left ventricle and a decrease in left ventricle's capacity to expand and fill with blood. As a result, there is a drop in the volume of blood in the left ventricle at the end of diastole, which leads to a decrease in the amount of blood pumped out by the heart with each beat thus reducing the overall efficiency of the heart. At the same time, it causes an increase in the central venous pressure [15]. When a patient changes from lying down to a face-down/prone position, there is often a reduction in stroke volume and cardiac output, which commonly results in a decrease in blood pressure.

In addition, prone positioning leads to a reduction of 30% to 35% in respiratory compliance and an elevation in peak airway pressure. This leads to a reduction in the amount of blood returning to the heart and the amount of blood pumped out by the heart [16]. Nevertheless, by using suitable cushioning to alleviate pressure on the thorax, it is possible to limit the decrease in respiratory compliance. However, hemorrhaging from vertebral veins is more noticeable when the patient is lying face down during spinal surgery. This can obstruct the surgeon's view, lengthen the operation, and lead to unstable blood circulation. The vertebrae are tightly associated with many venous plexuses that have thin walls and operate at low pressure. These plexuses are connected to the venous systems in the chest and abdomen. Elevated pressure results in heightened pressure on the vertebral veins, hence causing augmented bleeding during spinal surgery. When veins get filled with blood and arteries have low blood pressure, it can lead to inadequate blood flow to the spinal cord and possible tissue damage due to lack of oxygen [17].

Spinal hydatid cysts, although rare, can present significant challenges in management. Proper preoperative evaluation, anticipation, recognition, and management of potential intraoperative complications like anaphylactic reactions and a well-organized postoperative plan are essential in managing these complex cases and preventing recurrences.

## Conclusions

This highlights the crucial significance of examining primary spinal hydatid cysts as a potential cause of spinal cord compression syndrome, especially in regions where the disease is prevalent. Timely surgical intervention, together with the use of further anthelminthic therapy, is crucial for properly managing this condition and reducing the likelihood of recurrence. However, an anesthesiologist may face several difficulties while dealing with this morbidity, especially when a patient is positioned prone for surgery and particularly when cysts break during removal. This can make it challenging to identify and handle anaphylaxis while the patient is under general anesthesia. The primary objective is to ascertain the fundamental characteristics of hydatid cyst illness, discuss the anaesthetic issues, and provide guidance on preventing and managing anaphylaxis during surgery performed under general anaesthesia.
